# Editorial: Sex and Gender Differences in Body Image

**DOI:** 10.3389/fpsyg.2019.01696

**Published:** 2019-07-18

**Authors:** Andrea S. Hartmann, Elizabeth Rieger, Silja Vocks

**Affiliations:** ^1^Institute of Psychology, Clinical Psychology and Psychotherapy, Osnabrück University, Osnabrück, Germany; ^2^Research School of Psychology, Australian National University, Canberra, ACT, Australia

**Keywords:** sex, gender, body image, body image disturbance, sexual orientation, gender identification

Body image and its disturbance (BID) are multidimensional constructs that incorporate perceptual, cognitive, affective, and behavioral components regarding one's physical appearance (e.g., shape/weight) and function (e.g., health) (Pruzinsky and Cash, 2002). There is a perceptual component entailing a misperception of one's body (e.g., size) (e.g. Vocks et al., 2007). Additionally, there is a cognitive-affective component that includes dysfunctional processing of body-specific information (e.g., attention) (Aspen et al., 2013), and negative body-related thoughts, emotions, and attitudes toward one's own body (Vocks et al., 2007; Hrabosky et al., 2009). Lastly, BID can be captured on a behavioral level as checking, avoidance, or investment in the body (Hrabosky et al., 2009; Nikodijevic et al., 2018).

These three components are incorporated in the model of BID by Cordes et al. (2015). The model, furthermore, stresses the relevance of biopsychosocial factors in the development and maintenance of BID. For example, the model highlights the role of peer groups (e.g., teasing), the media, family, and cultural influences (e.g., gender roles) as well as biological factors (e.g., hormones or sexual orientation) that are theorized to exert a negative influence on body image through the internalization of body ideals and social comparison processes. Yet, research on the influence of gender, sex, gender identification, and sexual orientation on the various components of BID, and the relevant biopsychosocial factors, is scarce. As such, the aim of the present special issue is to address this gap by compiling studies in which gender, sex, gender identification, and sexual orientation are related to body image and its disturbance in various population using diverse research methods.

Three articles in this special issue shed light on gender differences in specific component or influential biopsychosocial factors. The first paper targets gender differences in affective and cognitive responses to various forms of mirror checking behavior (Tanck et al.). This experimental study showed that both checking of positively or negatively valenced body parts led to a significant increase in negative affect. Furthermore, it was shown that in both men and women, eating pathology predicted negative affect immediately after a checking episode. However, irrespective of checking positively or negatively valenced body parts, gender differences emerged, pointing to a higher body satisfaction in men. The authors conclude that body satisfaction in men might be more positive and more stable, but that pathological eating behavior renders both genders more vulnerable to checking which in turn is detrimental for affect and therewith might maintain the behavior. The second contribution (Schmidt and Martin) focuses on teasing experiences as one factor contributing to the development of BID. Results from this study indicated that the extent of teasing experiences was comparable across genders and was positively related to appearance-based rejection sensitivity and dysmorphic concerns in men and women. However, these experiences impacted on general mental health outcomes only in women, mediated by appearance-based rejection sensitivity and dysmorphic concerns. The last of these three articles (Voges et al.) focuses on the identification bias, a facet of distorted cognitive information processing. Findings from this experimental study indicated that women did not show any self-serving double standards, and also showed fewer self-deprecating double standards compared to men. The ability of men to self-enhance in the context of desirable bodies might foster body satisfaction.

Two further papers focus on body-related information processing in females specifically. Gledhill et al. concentrate on perception distortion when viewing bodies among female patients with anorexia nervosa and controls. Participants estimated bodies of other females, and the findings indicated that both groups tended to overestimate bodies with a low body mass index (BMI) and underestimate bodies with a high BMI, an effect known as the contraction effect (Cornelissen et al., 2013). This suggests that overestimation of one's own underweight body in anorexia nervosa stems from attitudinal rather than perceptual factors, particularly in cases when the body that is being judged approaches normal weight or above. The second article looks into how attentional bias is influenced by the menstrual cycle (Krohmer et al.). The researchers had women participate in a free-viewing eye-tracking experiment presenting their own body, once during their ovulation and once in the late luteal phase. During ovulation, participants felt more attractive and showed less attention to disliked body parts than during the late luteal phase, while a control group on hormonal contraceptives did not differ in their ratings across the two points of their menstrual cycle. This study highlights the potential contribution of a neglected biological factor to BID.

Two further contributions focus on body image in individuals with different sexual orientations and/or gender identities. First, Moreno-Dominguez et al. surveyed heterosexual, lesbian, and bisexual women with regard to the association between body satisfaction and sexual satisfaction. Body concerns were found to be less related to sexual satisfaction in lesbian compared to hetero- and bisexual women, suggesting an attenuation in the influence of body ideals in women's sexual satisfaction, possibly contributed to by the absence of the “male gaze”. Second, Bell et al. investigated eating disorder symptoms and proneness in gay men, lesbian women, and transgender and nonconforming (TGNC) adults. Findings indicated that lesbian women and TGNC adults show a higher proneness for eating disorders than gay men. Mediation analyses showed that thwarted belongingness and perceived stigma had an indirect association with eating disorder proneness mediated by self-compassion and depression in gay men, depression in lesbian women, and self-compassion in TGNC adults. The authors conclude that the interpersonal theory of eating disorders extends to sexual minority and gender diverse populations, but also highlight the additional relevance of stigma and self-compassion to potentially mitigate the impact of this stigma.

Overall the findings yield novel information regarding the development and maintenance of positive body image and BID, which can help to foster refinements in the theoretical frameworks. These more nuanced frameworks that are sensitive to gender, sex, gender identification, and sexual orientation can then be used to inform research, with the ultimate aim of yielding more tailored, and hence effective, BID interventions.

## Author Contributions

AH drafted the manuscript. ER and SV critically reviewed and edited it. All authors approved the final manuscript.

### Conflict of Interest Statement

The authors declare that the research was conducted in the absence of any commercial or financial relationships that could be construed as a potential conflict of interest.
